# Event-related potential: An overview

**DOI:** 10.4103/0972-6748.57865

**Published:** 2009

**Authors:** Shravani Sur, V. K. Sinha

**Affiliations:** Department of Psychiatry, RINPAS, Ranchi, India; 1 Department of Psychiatry, CIP, Ranchi, India

**Keywords:** Event-related potential, Psychiatric disorders, Neurotransmission

## Abstract

Electroencephalography (EEG) provides an excellent medium to understand neurobiological dysregulation, with the potential to evaluate neurotransmission. Time-locked EEG activity or event-related potential (ERP) helps capture neural activity related to both sensory and cognitive processes. In this article, we attempt to present an overview of the different waveforms of ERP and the major findings in various psychiatric conditions.

Richard Caton (1842–1926), a medical lecturer at Liverpool, was the pioneer in the field of evoked potential. He observed that “feeble currents of varying direction pass through the multiplier when the electrodes are placed on two points of the external surface.” This sentence marked the birth of the electroencephalogram (EEG), though it was invented much later by Hans Berger, a German Psychiatrist, in 1929.

## WHAT IS EVENT-RELATED POTENTIAL?

Event-related potentials (ERPs) are very small voltages generated in the brain structures in response to specific events or stimuli (Blackwood and Muir, 1990). They are EEG changes that are time locked to sensory, motor or cognitive events that provide safe and noninvasive approach to study psychophysiological correlates of mental processes. Event-related potentials can be elicited by a wide variety of sensory, cognitive or motor events. They are thought to reflect the summed activity of postsynaptic potentials produced when a large number of similarly oriented cortical pyramidal neurons (in the order of thousands or millions) fire in synchrony while processing information (Peterson *et al*., 1995). ERPs in humans can be divided into 2 categories. The early waves, or components peaking roughly within the first 100 milliseconds after stimulus, are termed ‘sensory’ or ‘exogenous’ as they depend largely on the physical parameters of the stimulus. In contrast, ERPs generated in later parts reflect the manner in which the subject evaluates the stimulus and are termed ‘cognitive’ or ‘endogenous’ ERPs as they examine information processing. The waveforms are described according to latency and amplitude.

## DIFFERENT ERP WAVEFORMS

### P50 wave

The amount of attenuation in the neural response to the second of the two identical stimuli indexes the strength of the inhibitory pathway. This paradigm has been adapted as a test of sensory gating principally through the study of the P50 waveform. Sensory gating is crucial to an individual’s ability to selectively attend to salient stimuli and ignore redundant, repetitive or trivial information, protecting the brain from information overflow (Light and Braff, 2003). The most positive peak between 40 and 75 msec after the conditioning stimulus is the P50 (Olincy *et al*., 2005). The P50 amplitude is the absolute difference between the P50 peak and the preceding negative trough (Clementz *et al*., 1997). P50 can be elicited by either the “paired click” paradigm or the “steady-state” paradigm.

### N100 or N1 wave

A negative deflection peaking between 90 and 200 msec after the onset of stimulus, is observed when an unexpected stimulus is presented. It is an orienting response or a “matching process,” that is, whenever a stimulus is presented, it is matched with previously experienced stimuli. It has maximum amplitude over Cz and is therefore also called “vertex potential.”

### P200 or P2 wave

P200 or P2 wave refers to the positive deflection peaking around 100-250 msec after the stimulus. Current evidence suggests that the N1/P2 component may reflect the sensation-seeking behavior of an individual.

### N200 or N2 wave

Is a negative deflection peaking at about 200 msec after presentation of stimulus.

There are 3 components of the N200 waveform —

#### N2a/ Mismatch negativity (MMN)

MMN is a negative component which is elicited by any discriminable change (Näätänen and Tiitinen, 1998) in a repetitive background of auditory stimulation (Winkler *et al*., 1996). MMN represents the brain’s automatic process involved in encoding of the stimulus difference or change.

#### N2b

It is slightly later in latency than the N2a and appears when changes in physical property of the stimulus are task relevant.

#### N2c

It is the classification N2, elicited when classification of disparate stimuli is needed.

### N300

N300 is a recent finding in the context of semantic congruity and expectancy.

### P300

The P3 wave was discovered by Sutton *et al*. in 1965 and since then has been the major component of research in the field of ERP. For auditory stimuli, the latency range is 250-400 msec for most adult subjects between 20 and 70 years of age. The latency is usually interpreted as the speed of stimulus classification resulting from discrimination of one event from another. Shorter latencies indicate superior mental performance relative to longer latencies. P3 amplitude seems to reflect stimulus information such that greater attention produces larger P3 waves. A wide variety of paradigms have been used to elicit the P300, of which the “oddball” paradigm is the most utilized where different stimuli are presented in a series such that one of them occurs relatively infrequently — that is the oddball. The subject is instructed to respond to the infrequent or target stimulus and not to the frequently presented or standard stimulus. Reduced P300 amplitude is an indicator of the broad neurobiological vulnerability that underlies disorders within the externalizing spectrum {alcohol dependence, drug dependence, nicotine dependence, conduct disorder and adult antisocial behavior} (Patrick *et al*., 2006).

### N400

It is a negative wave first described in the context of semantic incongruity, 300–600 msec post-stimulus (Kutas and Hillyard, 1980). N400 is inversely related to the expectancy of a given word to end a sentence.

### P600

In the domain of language processing, a P600 effect occurs to sentences that (a) contain a syntactic violation, (b) have a nonpreferred syntactic structure or (c) have a complex syntactic structure (Osterhout and Holcomb, 1992).

### Movement-related cortical potentials

MRCPs denote a series of potentials that occur in close temporal relation with movement or movement-like activity. These may occur before/during or after the movement and they refer to the associated preparedness for movement in the cortex. Kornhuber and Deecke (1965) distinguished 4 components of the MRCPs, viz., (1) Bereitschafts potential, (2) Reafferent potential, (3) Pre-motion positivity and (4) Motor potential.

### Contingent negative variation

Richard Caton in 1875 first used the term *negative variation* while describing electrical activity of gray matter, while Walter (1964) coined the term *contingent negative variation (CNV)*. CNV can be elicited by a standard reaction time paradigm (S1-S2-motor response) or only by paired stimuli without any motor response (S1-S2 paradigm). A first stimulus (S1) serves as a preparatory signal for an imperative stimulus (S2) to which the subject must make a response. In the S1-S2 interval, there are early and late CNV components. Early CNV is considered as indicator of arousal processes, and late CNV is associated with attention to the experimental task.

### Post-imperative negative variation

PINV is the delay in CNV resolution, that is, negativity continues after S2. PINV is a marker of sustained cognitive activity.

## EVENT-RELATED POTENTIAL CHANGES IN PSYCHIATRIC DISORDERS

### Alcohol dependence syndrome

#### N1 P2

Alcohol-induced attenuation of N1 and P2 amplitudes has been consistently reported. The N1 amplitude was dose-dependently suppressed by alcohol, and the N1 peak latency was prolonged by the higher (0.85 g/ kg) dose of ethanol, thus supporting the previous observations.

#### N2

There is increase in the latency of N200.

#### P300

Acute ethanol intake is seen to reduce P300 amplitude. However, wave abnormalities have also been seen in abstinent individuals and in the first-degree relatives of patients (Patrick *et al*., 2006).

#### CNV, MRCP

Decreased amplitude of CNV and MRCP denoting deficits in executive functioning in alcoholic patients has been reported.

### Schizophrenia

#### P300

One of the most robust neurophysiological findings in schizophrenia is decrease in P300 amplitude. P300 is often smaller in amplitude and longer in latency in patients who have been ill longer. P300 latency was found to be increased in schizophrenic patients but not in their first-degree relatives (Simlai& Nizamie, 1998). In longitudinal analyses, P300 amplitude is sensitive to fluctuations in the severity of positive symptoms, independent of medication, and to the enduring level of negative symptom severity (Mathalon *et al*., 2000).

#### P50

Diminished P50 suppression has been reported in patients with schizophrenia (Bramon *et al*., 2004) and in their non-psychotic relatives (Clementz *et al*., 1998).

#### N1, P2, N2

Schizophrenia patients have demonstrated reduced N100, P200 and N200 amplitudes (O’Donnell *et al*., 2004).

#### MMN

There is decreased MMN amplitude, as well as abnormal MMN topographical distribution, in treatment-refractory patients with schizophrenia (Milovan, 2004).

#### CNV, BP

CNV amplitude was noted to be shorter and latency longer, localized to the left central region, in schizophrenia patients. BP interval and amplitude were found to be increased when compared to controls (Simlai& Nizamie, 1998). BP latency was found to be decreased in patients denoting impairment in planning movement and decision making (Duggal& Nizamie, 1998).

#### Late components

There is an increase in N400 and P600 latencies in schizophrenic patients.

### Bipolar affective disorder

#### P50

P50 suppression deficits have been found in patients with bipolar disorder with psychotic symptoms, as well as in their unaffected first-degree relatives, suggesting P50 to be an endophenotypic marker for the illness (Schulze *et al*., 2007).

#### P300

Salisbury *et al*. (1999) have recently noted P300 reduction in manic psychosis. Latency prolongation and amplitude reduction were seen in chronic bipolar patients (O’Donell *et al*., 2004).

### Depression

#### P300

Reduced amplitude of P300 has been seen in depressed patients, mainly with suicidal ideations, psychotic features or severe depression (Hansenne *et al*., 1996).

## NEUROTIC DISORDERS

### Phobia

#### P300

Studies show that individuals with spider and snake phobias showed significantly larger P300 amplitudes than healthy controls when exposed to pictures of their feared objects, indicating enhanced processing of stimuli that reflect critical fear concerns (Miltner *et al*., 2000).

### Panic disorder

#### P300

An enlarged frontal P3a to distractor stimuli among patients has been reported using a three-tone discrimination task, supporting the hypothesis of dysfunctional prefrontal-limbic pathways. In addition, a longer P3b latency in drug-free patients than in unaffected controls has also been reported as possible evidence of a dysfunctional hippocampus and amygdala (Turan *et al*., 2002).

### Generalized anxiety disorder

ERPs elicited by threat-relevant stimuli support the existence of an attentional bias, showing larger amplitude of P300 and slow waves in response to fear-related words or pictures in subjects with high-trait anxiety or anxiety disorders when compared with healthy controls (De Pascalis *et al*., 2004).

### Obsessive compulsive disorder

#### N2, P3

OCD patients are seen to have significantly shorter P300 and N200 latencies for target stimuli and greater N200 negativity when compared with normal controls. However, there are no significant relationships between these ERP abnormalities in OCD patients and the type or severity of their OCD symptoms. Paul and Nizamie (1999) found increased P300 latency in OCD patients but no difference in amplitude.

### Posttraumatic stress disorder

#### P50

There are reports of a reduction of the P50 suppression response in persons with posttraumatic stress disorder (PTSD) (Neylan *et al*., 1999; Skinner *et al*., 1999).

#### P300

The most common finding is reduced P300 amplitudes (Metzger *et al*., 1997).

### Dissociative disorder

#### P300

Patients showed significant reduction in the amplitudes of P300 during dissociative disorders compared with the levels at remission. The latency of P300 remained unchanged. The amplitudes of P300 might be a state-dependent biological marker of dissociative disorders.

### Personality disorders

In healthy subjects, several studies have reported some relationships between N200, P300 and personality. A consistent result of these studies is that introverts exhibit higher P300 amplitude than extroverts. P300 amplitude is weakly correlated (positively) to the self-directedness dimension; and CNV, to cooperativeness. Longer N200 latency may be associated with higher harm avoidance score. N200 amplitude is negatively correlated to persistence. This indicates that lower N200 amplitude may be related to a higher persistence score.

## CONCLUSION

ERP constitutes a millisecond-by-millisecond record of neural information processing, which can be associated with particular operations such as sensory encoding, inhibitory responses and updating working memory. Thus it provides a noninvasive means to evaluate brain functioning in patients with cognitive disorders and is of prognostic value in few cases. ERP is a method of neuropsychiatric research which holds great promise for the future.

## References

[1] Blackwood D. H. R, Muir W. J (1990). Cognitive brain potentials and their application. The British Journal of Psychiatry.

[2] Bramon E, Rabe-Hesketh S, Shama P (2004). Metaanalysis of the P300 and P50 waveforms in schizophrenia. Schizophrenia Research.

[3] Clementz B. A, Geyer M. A, Braff D. L (1997). P50 suppression among schizophrenia and normal comparison subjects: A methodological analysis. Biological Psychaitry.

[4] Clementz B. A, Geyer M. A, Braff D. L (1998). Poor P50 suppression among schizophrenia patients and their first-degree biological relatives. American Journal of Psychiatry.

[5] De Pascalis V, Strippoli E, Riccardi P (2004). Personality, event-related potential (ERP) and heart rate (HR) in emotional word processing. Pers Individ Diff.

[6] Hansenne M, Pitchot W, Gonzalez-Moreno A (1996). Suicidal behavior in depressive disorder, an event related potential study. Biological Psychiatry.

[7] Kornhuber H. H, Deecke L (1978). An electrical sign of participation of the mesial “supplementary” motor cortex in human voluntary finger movements. Brain Research.

[8] Kutas M, Lindamood T, Hillyard S, Kornblum S, Requin J (1984). Word probability and event related potentials during sentence processing. In Preparatory states and processes.

[9] Light G. A, Braff D. L (2003). Sensory gating deficits in schizophrenia, Can we parse the effects of medication, nicotine use, and changes in clinical status?. Clinical Neuroscience Research.

[10] Metzger L. J, Orr S. P, Lasko N. B (1997). Evidence for diminished P3 amplitudes in PTSD. Ann N Y Acad Sci.

[11] Miltner W. H. R, Krieschel S, Gutberlet I (2000). P300–a signature for threat processing in phobic subjects. Psychophysiology.

[12] Milovan D. L, Baribeau J, Roth R. M (2004). ERP study of pre-attentive auditory processing in treatmentrefractory schizophrenia. Brain Cogn.

[13] Näätänen R, Tiitinen H, Sabourin M, Craik F.I.M, Robert M (1998). Auditory information processing as indexed by the mismatch negativity. Advances in Psychological Science. Biological and cognitive aspects, 2, Psychology Press/Erlbaum (UK) Taylor and Francis, Hove, UK.

[14] Neylan T. C, Fletcher D. J, Lenoci M (1999). Sensory gating in chronic posttraumatic stress disorder: Reduced auditory P50 suppression in combat veterans. Biol Psychiatry.

[15] O’Donnell S, Hesselbrock V, Tasmann A (1987). P3 amplitudes in two different tasks are decreased in young men with a history of paternal alcoholism. Alcohol.

[16] O’Donnell B. F, Vohs J. L, Hetrick W. P (2004). Auditory event-related potential abnormalities in bipolar disorder and schizophrenia. International Journal of Psychophysiology.

[17] Olincy A, Martin L (2005). Diminished suppression of the P50 auditory evoked potential in bipolar disorder subjects with a history of psychosis. American Journal of Psychiatry.

[18] Osterhout L, Holcomb P. J (1992). Event related brain potentials elicited by syntactic anomaly. Journal of Memory and Language.

[19] Patrick C. J, Bernat E. M, Malone S. M, Iacono W. G, Krueger R. F, McGue M (2006). J., Bernat, E. M.

[20] Peterson N.N, Schroeder C. E, Arezzo J. C (1995). Neural generators of early cortical somatosensory evoked potentials in the awake monkey. Electroencephalogrphy and Clinical Neurophysiology.

[21] Salisbury D. F, Shenton M. E, McCarley R. W (1999). P300 topography differs in schizophrenia and manic psychosis. Biological Psychiatry.

[22] Schulze K. K, Hall M. H, McDonald C (2007). P50 auditory evoked potential suppression in bipolar disorder patients with psychotic features and their unaffected relatives. Biol Psychiatry.

[23] Simlai J, Nizamie S. H (1998). Event related potentials-P300, CNV, MRCP in drug naïve and drug free schizophrenia. Thesis submitted to Ranchi University, in partial fulfillment of requirement of degree of doctor of medicine in psychiatry.

[24] Skinner R. D, Rasco L. M, Fitzgerald J (1999). Reduced sensory gating of the P1 potential in rape victims and combat veterans with posttraumatic stress disorder. Depress Anxiety.

[25] Sutton S, Braren M, Zubin J (1965). Evoked potential correlates of stimulus uncertainty. Science.

[26] Turan T, Esel E, Karaaslan F (2002). Auditory event-related potentials in panic and generalised anxiety disorders. Biological psychiatry.

[27] Walter, W. G (1964). Slow potential waves in the human brain associated with expectancy, attention and decision. Arch Psychiatr Nervenkr.

[28] Winkler I, Karmos G, Näätänen R (1996). Adaptive modeling of the unattended acoustic environment reflected in the mismatch negativity event-related potential. Brain Research.

